# Foxp3 expression in breast cancer patient from Qatar: survival analysis

**DOI:** 10.1186/2051-1426-3-S1-O2

**Published:** 2015-08-14

**Authors:** Mahmoud G Mohamed, Hina Sarwath, Shaykha Alqahtani, Salha Bujjasoum, Imad Bin Mujeeb, Mufeed Almesteri, Hikmat Bugrein, Prem Chandra, Shahinaz Bedri

**Affiliations:** 1 Research, Weill Cornell Medical College in Qatar, Doha, Qatar; 2 Hamad Medical Corporation, Doha, Qatar

## 

In breast cancer, the presence of Foxp3 (Tregs) [1] within the tumor milieu has been a matter of debate. Some studies have determined that infiltration of Tregs was associated with poor survival, while others revealed no impact on survival, however this depends on their type, type of cells expressing Foxp3 and the density of the Tregs population [2].

The goal of our study was to quantify Foxp3 in breast cancer patients from Qatar and correlate with their survival.

## Methodology

Expression of FoxP3 was studied in 132 FFPE samples with known clinico-pathological data by immunohistochemistry technique and quantified by modified H-score system by pathologist. Results were analyzed via SPSS.

## Results

Analysis was carried for 132 patients. Age at time of diagnosis was 49 ±10.4 years. 76.2% of the patients showed positive expression of FoxP3. FoxP3 expression was not correlated with patient age or hormone receptors. Expression of Foxp3 positively correlate with better patient survival when compared to negative expression (94.1, 95% CI 85.6 - 102.6 versus 83.6, 95% CI 71.8 - 95.5, p 0.60).

## Conclusion

FoxP3 is expressed on lymphocytes that are present in the tumor microenvironment regardless of breast cancer subtypes. Foxp3 is correlated with better survival.
